# HD Physiology Project—Japanese efforts to promote multilevel integrative systems biology and physiome research

**DOI:** 10.1038/s41540-016-0001-0

**Published:** 2017-01-16

**Authors:** Kazuharu Furutani, Kunichika Tsumoto, Yoshihisa Kurachi, for HD physiology project investigators

**Affiliations:** 1 Department of Pharmacology, Graduate School of Medicine, Osaka University, Osaka, 565-0871 Japan; 2 Global Center for Medical Engineering and Informatics, Osaka University, Osaka, 565-0871 Japan; 3 HD Physiology Project, Osaka, Japan

## Abstract

The HD Physiology Project is a Japanese research consortium that aimed to develop methods and a computational platform in which physiological and pathological information can be described in high-level definitions across multiple scales of time and size. During the 5 years of this project, an appropriate software platform for multilevel functional simulation was developed and a whole-heart model including pharmacokinetics for the assessment of the proarrhythmic risk of drugs was developed. In this article, we outline the description and scientific strategy of this project and present the achievements and influence on multilevel integrative systems biology and physiome research.

## Introduction

Systems biology is the field of study that considers the complexity of biological systems among different scales of biological organization, from the molecular to the cellular, tissue, organ, organism, and even societal and ecosystem levels.^1^ Systems biology aims for a more profound understanding of biological processes and is characterized by the development of experiment-based mathematical modeling and its application, combined with experimental empirical studies. Therefore, systems biological approaches rely heavily on experimental and quantitative data, as well as modeling techniques spanning multiple spatial and temporal scales and also different levels of biological organization.

The term “*physiome*,” from “*physio*-” (life) and “-*ome*” (as a whole), comprehensively and quantitatively describes the physiological dynamics of the functional behaviors of organisms, including humans, and is built upon information and structure (i.e., the genome, proteome, and morphome). Global efforts have led to the establishment of various national projects on the physiome, such as the German virtual liver network (http://www.virtual-liver.de/). Another physiome project is the Wellcome Trust Heart Physiome Project (Physiome Project, http://physiomeproject.org/), which is part of the wider International Union of Physiological sciences (IUPS) physiome project.^2,3^ This IUPS physiome project aims to completely describe the heart, including its anatomy; molecular, cellular, and organ mechanics; electrophysiology; electro-mechanical coupling; and metabolic processes in health and disease states. In addition, there are several scientific societies, for example, the Cardiac Physiome Society (http://cardiacphysiome.org/) that was created to respond to the researcher’s need and hold an academic meeting to discuss the current research topics and to exchange ideas cross-nationally in the field of the integrative systems biology.

The massive amount of existing biological data obtained in a variety of contexts are subdivided and fragmented. This unwieldy amount of information makes it difficult to see the big picture of functional behaviors in biological systems. Life science researchers are confronting this issue. We should develop a framework for organizing knowledge, specifically that obtained from different levels, as well as methodology to study the dynamic behavior of complex biological systems.

## The HD Physiology Project

The HD Physiology Project (http://hd-physiology.jp/) whose name represents high-definition, human disease, and hierarchical-designed physiology, was a major Japanese project organizing a consortium of laboratories renowned for their expertise in heart physiology and circulatory molecular dynamics, thus encompassing the historical core of systems biology research in Japan. The project, with Japanese Ministry of Education, Culture, Sports, Science and Technology (MEXT) support (around ¥300,000,000 per year) from 2010 to 2016, tackled one of the major challenges in multilevel integrative systems biology and physiome. This interdisciplinary project focused on biological relativity between different organism levels and explored the systems theory for function emergence owing to the interactions among components. The consortium formed a strong network that shared expertise, tools, and methods, consequently enhancing synergy. Furthermore, to understand basic concepts of life, novel methodologies were required to integrate parts across multiple levels of organisms and new infrastructure was required for multilevel integrative systems biology and physiome research. During the 5 years of this project, a software platform for multilevel functional simulation with hierarchical-designed models was developed and a whole-heart model that included a pharmacokinetics model enabling assessment of the proarrhythmic risk of drugs was attempted. Furthermore, an integrative and holistic approach was introduced in order to reveal physiological and pathophysiological mechanisms. The ultimate goal was to identify key signaling molecules as novel drug targets for therapeutic intervention and/or as diagnostic biomarkers. Moreover, the consortium attempted to provide technical and theoretical frameworks for the HD Physiology Project and to lay the foundation for “predictive medicine.”

## Description and scientific strategy of the HD Physiology Project

In order to achieve its objectives, the 5-year program had to address several organizational challenges arising from the integration of multiple disciplines including molecular and cellular biology, mathematics, physics, computer science, tissue engineering, and clinical imaging. The program encompassed about 40 sub-projects and involved approximately 250 scientists including about 50 PIs/full professor and 150 junior faculties throughout Japan (Table 1). This organization was planned by the leading scientists to respond to the Japanese researcher’s need and was approved by the grants reviewing committee in 2010. Scientists involved in the planning had served as a dedicated core team of project managers after the launch. They held the general meeting twice a year to get all members together so that they had grasped the overall as well as individual situation. Then, as needed, they gave the members adequate advice, introduced other members who could help to solve the problem, and created the task force team to work on the issue. For example, they created a specialized task force team for the issues about model repository and huge computation of hierarchical designed physiology models. They also held the international symposium on this research three times to provide an opportunity for Japanese researchers inside and outside the project to discuss the current research topics and to exchange ideas cross-nationally in the field of integrative systems biology. As such, this project may provide insights into the challenges of managing large and complex biological science programs to ensure the delivery of ambitious objectives. Thus, our experience provides a blueprint for other major flagship programs.

**Table 1:** HD Physiology Project member list

HD Physiology Project				
Axis	Sub-project leader	Affiliation	Primal research area	
Axis 01	Yoshihisa Kurachi	Osaka University	Physiome and systems biology (cardiac system)	PL, CM
	Hiroaki Kitano	Okinawa Institute of Science and Technology Graduate University	Systems biology (software, tools)	PM, CM
	Akira Amano	Ritsumeikan University	Computational physiology (cardiovascular system)	CM
	Kengo Kinoshita	Tohoku University	Molecular dynamics simulation	CM
	Yoshiyuki Hari	Osaka University	Organic chemistry	CM
	Kazuhiro Aoki	Kyoto University	Molecular imaging	
	Yuji Imaizumi	Nagoya City University	Molecular biology	
	Akira Funahashi	Keio University	Computational technology	
	Kenichi Hagihara	Osaka University	Parallel computation	
	Hideki Oka	Tokai University	Structural biomechanics (tissue/organ)	
Axis 02	Ryuji Inoue	Fukuoka University	Physiology (ion channel, remodeling)	PM, CM
	Naomasa Makita	Nagasaki University	Cardiology (gene, arrhythmias)	CM
	Yoshihiro Ishikawa	Yokohama City University	Medical biology (G-protein signal transduction)	CM
	Haruo Honjo	Nagoya University	Biophysics (cardiac conduction system)	CM
	Kazuo Nakazawa	National Cerebral and Cardiovascular Center	Biomedical engineering (large scale heart simulation)	CM
	Kyoichi Ono	Akita University	Physiology (cardiac autorhythmicity)	
	Junko Kurokawa	Tokyo Medical and Dental University	Pharmacology (sex and gender aspects in arrhythmia)	
	Tomohiro Numata	Kyoto University	Physiology (ion channel)	
	Daiju Yamazaki	Kyoto University	Toxicology (Ca^2+^ signal)	
	Nagomi Kurebayashi	Juntendo University	Pharmacology (Ca^2+^ signal)	
	Satoshi Kurihara	The Jikei University School of Medicine	Physiology (muscle contraction)	
	Susumu Minamisawa	The Jikei University School of Medicine	Tissue engineering (Blood vessel)	
	Satomi Kita	Fukuoka University	Pharmacology (transporter)	
	Koichi Nakajo	National Institute for Physiological Sciences	Physiology (ion channel)	
	Takashi Aiba	National Cerebral and Cardiovascular Center	Clinician (gene, arrhythmias)	
	Haruo Ogawa	University of Tokyo	Structural biology (pump, transporter)	
	Hideki Ito	Shiga University of Medical Science	Clinician (gene, arrhythmias)	
	Ayako Takeuchi	Fukui University	Physiology (Ca^2+^ signal)	
	Tetsuo Shioi	Kyoto University	Cardiology (metabolic activity)	
	Yoichiro Kusakari	The Jikei University School of Medicine	Cardiology (heart failure)	
	Yasutaka Kurata	Kanazawa Medical University	Cardiology (triggered activities, bifurcation theory)	
	Motohiko Sato	Aichi Medical University	Biochemistry (G-protein signal transduction)	
Axis 03	Hiroshi Suzuki	University of Tokyo Hospital	Systems pharmacology and toxicology	PM, CM
	Fumiyoshi Yamashita	Kyoto University	Clinical pharmacology	PM, CM
	Yasushi Okuno	Kyoto University	Bioinformatics (drug discovery)	CM
	Hiroyuki Kagechika	Tokyo Medical and Dental University	Chemical biology (transcriptional factors, retinoids)	CM
	Sumio Ohtsuki	Tohoku University	Drug transport, targeted proteomics	CM
	Hiroyuki Kusuhara	University of Tokyo	Molecular pharmacokinetics	CM
	Toshiya Katsura	Kyoto University	Molecular pharmacokinetics	
	Yasuo Yoshioka	Osaka University	Drug delivery system	
	Shushi Nagamori	Osaka University	Biochemistry (proteomics, transporter)	
	Yuichiro Fujiwara	Osaka University	Physiology and structural biology (ion channel)	
	Sawako Tatsumi	Tokushima University	Nutrition science (Inorganic phosphate homeostasis)	
	Fumiaki Nin	Niigata University	Physiology (inner ear function)	
	Kazuo Matsubara	Kyoto University	Clinical pharmacology	
	Shigehiro Ohdo	Kyushu University	Pharmacotherapy (circadian rhythm)	
	Takeshi Hiyama	National Institute for Basic Biology	Neuroscience (body-fluid homeostasis)	

*PL* project leader, *PM* project manager, *CM* core member

**Fig. 1:**
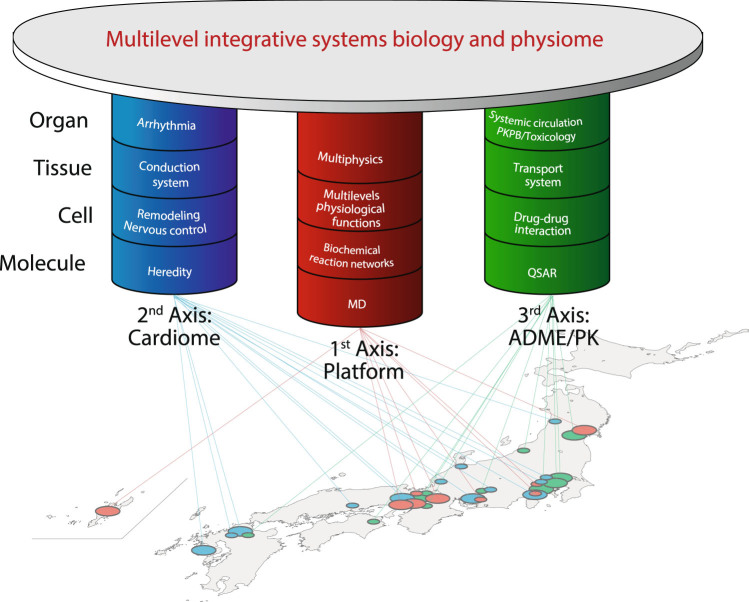
The HD Physiology Project framework research platform for multilevel systems biology, cardiome, and ADME/PK is being developed to understand basic concepts of life and general mechanisms of robustness and regulation of biological networks that hold true for different types of physiological issues

As illustrated in Fig. 1, the HD Physiology Project was organized in three primary research axes, with the motivation described later.

The first-axis, “Research and development of a software platform for integrative multilevel systems biology,” aimed to develop a better software platform for multilevel modeling and simulation in systems biology, as well as to provide novel technologies for accelerating calculations and for use in biological experiments such as molecular compounds and probes.

The second-axis, entitled “Multilevel systems biology of cardiac electrophysiological activity,” performed integrative research on cardiac action potential and excitation propagation, which underlie the heart’s function. This group was tasked with understanding the robustness of electrical activity in the heart on the basis of experimental studies from the molecular, cellular, tissue, and organ levels, as well as from the perspective of heart disease (e.g., arrhythmias).

The third-axis, entitled “Multilevel systems biology of small molecular dynamics in circulation,” investigated the systems that are homeostatically controlled by multiple organs. This group started with pharmacokinetics and investigated the crucial mechanisms underlying absorption, distribution, metabolism, and excretion (ADME) systems. In this project, we conducted physiologically based pharmacokinetic (PBPK) modeling for predicting the ADME of synthetic or natural chemical substances and hypothesis testing about the unknown mechanism underlying ADME, for example, lower-than-expected oral bioavailability.

The first axis was entrusted with a mission to provide new methodologies for studying multilevel biological phenomena that would benefit the rest of the consortium, as well as the fields of systems biology, physiology, pharmacology, and medicine. Testing the new methodological approaches in an iterative manner necessitated specific and explicit physiological issues with quantitative experimental verifications. Therefore, the second and third-axes were specialized to investigate cardiology and pharmacokinetics. The several important issues in cardiology arise from vertical integration in the single organ, i.e., heart, while those in pharmacokinetics arise from horizontal integration in the multiple organs. By adopting such different technical approaches and sharing the results, each axis expanded its own interests to other physiological and pathological issues toward the shared goal of quantitative theoretical and experimental investigation. Indeed, exchanges of materials such as specially synthesized chemical compounds, genes, model animals/cells, biological structural data, and physiological hierarchy markup language (PHML) models and techniques, including building/simulation of physiological models, quantitative experimental techniques, and mathematical analyses with the software tools, as well as formal collaboration, occurred across several groups within the network of the consortium. A large number of researchers of this project have contributed to construct the flagship model that integrates the knowledge about electrocardiology and pharmacokinetics on the platform in order to guide the development of software framework, which further promotes the researches in the 2nd and third-axes. By promoting the research cycle, we expected to identify general mechanisms of robustness and regulation of biological networks that hold true for different types of physiological issues.

## Research achievements

### Research and development of a software platform for integrative multilevel systems biology (first-axis)

**Fig. 2:**
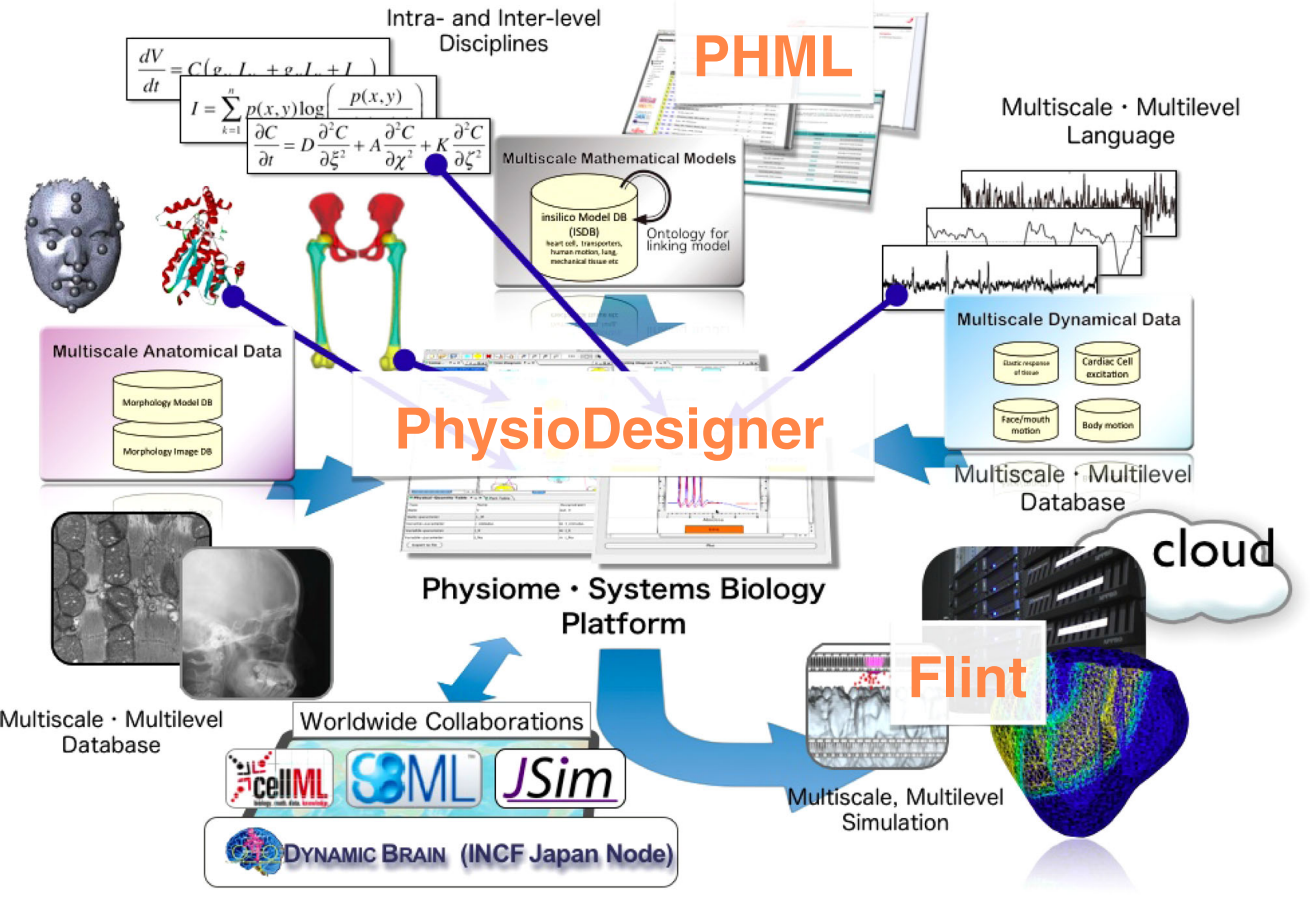
Information flow with a focus on PhysioDesigne. This illustration is reproduced and reprinted with permission from the author and from the website Physiome.jp (http://physiome.jp/)

When the project was started, information on the subcellular and cellular levels could be expressed by the model descriptive language, systems biology markup language (SBML), which became the de facto standard of the field of systems biology.^4^ Accordingly, mathematical models described in SBML enabled the calculation of the dynamic behaviors of complex biomolecular networks and pathways by using CellDesigner software.^5,6^ Next, a descriptive language that can handle physiological information from multiple levels (including the tissue, organ, and organism levels) was required. Therefore, we developed a new XML-based descriptive language format called PHML to model multilevel physiological systems. In PHML, each of the elements constructing a model is called a module.^7–9^ Hence, a model is represented as an aggregate of modules. Structural and functional relationships among the modules are defined by edges. Each module is quantitatively characterized by several physical quantities that represent dynamical variables, constants, and time-dependent parameters. Morphological data and time series data can be assigned to physical quantities as well. Definitions of the dynamics or functions of physical quantities must be explicitly described by mathematical equations such as algebraic equations and ordinary differential equations (ODEs) that can include stochastic terms. Hence, when we try to describe conformational changes of, for example, tissues or body parts mathematically, such dynamical motions can be described in PHML. Partial differential equations (PDEs) are also available, but only with limited use, as a function to involve PDEs is still under development. Moreover, we developed PhysioDesigner, which is a platform on which users can build mathematical models of multilevel physiological systems with a graphical user interface, and Flint, which is a simulator developed concomitantly with PhysioDesigner (Fig. 2)^8,10^ (Physiome.jp, http://physiome.jp/). PhysioDesigner users can also develop mathematical models with high freedom to integrate other models of organs, tissues, and physiological functions, as PhysioDesigner is a versatile platform for physiome and integrative multilevel systems biology.

There have been pioneering efforts to develop modeling standards, such as SBML (The SBML.org, http://sbml.org/) for modeling mainly subcellular biochemical phenomena and CellML (http://physiomeproject.org/software/cellml)^11^ for physiological level modeling. PHML was developed to implement a unique scheme representing state-transition variables that take discrete states, such as close/open states of ionic channels, and to construct large-scale models based on template and instance modules. Moreover, collaborative functions with other descriptive languages were paid careful attention in PHML. PHML has a partial compatibility with CellML, i.e., a model written in CellML can be converted into PHML, and a model written using a part of PHML elements can be exported as a CellML model by PhysioDesigner. Besides, a module of PHML can import a whole SBML model, which is a unique feature of PHML to develop a multilevel model of physiological systems by hybridizing two different descriptive languages.

PhysioDesigner Version 1.0 α was launched in January 2012, and the latest version (1.5) was launched on 30 June 2016. PhysioDesigner has been downloaded approximately 1600 times in 50 countries from the project website, Physiome.jp (http://physiome.jp/). In addition, PhysioVisualizer and PHPlotter, which visualize the results of computer simulations, were implemented, and various databases, which is opened to the public, were built as a PH database (http://physiome.jp/phdb/index.html).

Although a large number of software programs for the arts and sciences have been developed, little attention has been paid to integration among programs or the resolution of inconveniences occurring when users use multiple programs.^12,13^ In order to respond to their needs, the Garuda Alliance (Garuda Platform, http://www.garuda-alliance.org/) was formed as a strategic partnership with software developers. Then, the subsequent platform that was developed was made available to researchers. The members of this HD Physiology Project have greatly contributed to the efforts. Now, domestic and foreign universities as well as research organizations expressed interest in this project. Approximately 80 programs currently support the Garuda Platform; these programs provide language independent Application Programming Interface to connect software as gadgets, and enable seamless integration among such programs intensively through the Garuda Gateway.

Meanwhile, other teams of the first-axis worked on cutting-edge methodologies and technologies such as molecular dynamics, coding, and parallel computing techniques, organic chemistry, and molecular imaging.

### Multilevel systems biology of cardiac electrophysiological activity (second-axis)

The second-axis focused on the function of a single organ, i.e., the heart, adopting a middle-out approach^14,15^ to examine functions at the molecular or tissue/organ level while investigating relatively well-known cardiomyocytes. In particular, the investigation of unique issues related to arrhythmia-associated genetic abnormalities, molecular remodeling, neural control, and cardiac excitation propagation aimed to understand the robustness or failure of electrical activity in the heart resulting from the vertical integration of functions evoked at each system level within the heart.

In order to advance the field of systems biology, we proposed novel ideas associated with the robustness of electrical activities in the heart, i.e., compensatory, protective, and prevent mechanisms. The first example (compensatory mechanism) resulted from simulations of the excitation conduction in ventricular myofiber models: electrical function evoked via the intercellular nanostructure between ventricular myocytes prevents conduction failure when the electrical coupling between myocytes through gap junctions is markedly reduced by diseases or drugs.^16,17^ The second example (protective mechanism) resulted from simulations of the action potential propagation in the two-dimensional network model of myocardial fibers within the atrioventricular (AV) node: an anatomical tissue architecture in the AV node can function to protect against conduction block-mediated arrhythmias.^18^ The third example (protective mechanism) resulted from simulations of the scroll-wave reentry in three-dimensional ventricular slab model: ventricular tissue architecture, including different types of myocyte and rotated fiber orientation, can reduce the risk of the development and sustainability of ventricular tachycardia/fibrillation attributed to the increase in the transmural dispersion of repolarization.^19^ The subcellular localization of signaling molecules and intra-/intercellular microstructures between the molecular and cellular levels as well as the tissue architecture comprising various types of cells among the cellular, tissue, and organs levels are closely associated with the robustness of electrical phenomena in the heart as a principle of complementarity. These theoretical predictions may be an example of the nature of biological system and help to understand how we should organize knowledge in multilevel systems biology.

Furthermore, by the comprehensive evaluation with experimental data and simulation analysis, we successfully provided the following new drug targets and strategies for improving arrhythmia: (1) gap junction openers for the treatment of progressive cardiac conduction defects;^20^ (2) a type 5 adenylyl cyclase selective inhibitor for the treatment of atrial fibrillation;^21–24^ (3) a hERG channel inhibitor with a facilitation action for protection against triggering activities;^25,26^ and (4) a TRPM4 selective inhibitor for the treatment of atrial and ventricular arrhythmias associated with elevated TRPM4 expression.^27,28^ Basically, each example was hypothesized from the extensive simulation studies with mathematical models of the etiology of the heart diseases and the pharmacology of drug candidates, and then evaluated and validated in the animal study. Of note, the type 5 adenylyl cyclase selective inhibitor is currently undergoing clinical study, because sufficient efficacy was already demonstrated in cellular and animal experiments as well as mathematical simulation studies.

### Multilevel systems biology of small molecular dynamics in circulation (third-axis)

The third-axis focused on the emergence of functions resulting from the interdependence of multiorgan functions, i.e., the horizontal integration of biological functions evoked at each organ level. As individual organs are connected by blood flow, the description and prediction of changes in the concentrations of small compounds in blood are the most important aspects for understanding the behavior of small molecules in each organ and tissue. From the viewpoint of the homeostasis of small molecules in the blood, the prototype beta version of whole-body physiological ADME (multi-compartment or lumped parameter ODE) models based on existing descriptions of pharmacokinetics on our platform was implemented; although incomplete, these models can roughly predict the behavior by in vitro-in vivo extrapolation and identify unresolved issues by complementing experimental and computational approaches. PhysioDesigner was used to model the abstracted organ modules as compartments within which the concentration of a drug is assumed to be uniformly equal and connected by circulation—not just in a mathematical model, but more importantly in a series of models that are linked across scales to represent organ function—and incorporate key reaction processes important for the function of the higher levels from the lower ones (e.g., cellular and molecular events). Incorporating quantitative data on ADME obtained in vitro^29–32^ into the physiological ADME model enabled the reproduction and prediction of the pharmacokinetics observed in humans in vivo even when drug–drug interactions occur.^33^ Modularity supported by PHML is adequate for this strategy and is not supported by other commercial or open-source software.

As PBPK models require many parameters to reproduce physiological functions, efficient parameter estimation methods are essential. We successfully applied a recently developed algorithm called the Cluster Newton Method (CNM) to estimate a feasible solution space.^34^ After improving the original CNM algorithm to maintain parameter diversities, a feasible solution space was successfully estimated for parameters in the PBPK model of irinotecan (a chemotherapy agent) to predict large inter-individual variability in drug responses in cancer patients. Application of the CNM achieved a feasible solution space by solving inverse problems of a system containing ODEsThis method may provide reliable insights into other complicated phenomena that have several parameters to estimate with limited information. This method is also helpful for designing prospective studies further investigating phenomena of interest.

As was the case in studies from the second-axis, those from the third-axis demonstrated the common concept in multilevel systems biology: the architecture of organs and tissue, including spatial tissue microstructure, is crucial for recapturing physiological phenomena at the individual level. For example, although several models for the prediction of local pharmacokinetics in the gastrointestinal tract have been reported, these models are incapable of analyzing nonlinear pharmacokinetics because they do not consider drug concentration in enterocytes, in which metabolism and transport processes among others are actually nonlinear. We successfully released a new local pharmacokinetic model of gastrointestinal absorption, the translocation model, which uses an anatomically relevant, minimally segmented structure and explains linear and nonlinear intestinal drug absorption, metabolism, and transport.^35^ The translocation model is relatively simple compared to existing models, making it useful for the prediction of drug absorption during drug development in the future. Furthermore, the physiological model would more accurately analyze various events occurring during the ADME process by adjusting the model’s structure and parameters as necessary. We also developed a technique for the absolute quantitative determination of the expression levels of multiple proteins, such as transporters and enzymes participating in medicine excretion and xenobiotic detoxification. Our comprehensive circulation model that incorporates the translocation model and the quantitative information obtained can accurately provide key information to determine the exposure of the drug to therapeutic targets in the body, while each model that works independently enables us to deepen our understanding about the detailed mechanism of PBPK.

Target occupancy profiles for drugs were calculated from their mean unbound plasma concentrations and reported dissociation constant (*K*_d_) values. Therefore, a computer simulation can identify the effects of drugs and highlight ways to maximize their clinical benefit and minimize side effects.^36^ This study focused on sunitinib, a multi-kinase inhibitor with antitumor and anti-angiogenic activities that is known to cause numerous adverse reactions.^37^ In silico simulations indicated that unwanted drug-mediated inhibition of phosphorylase kinase might cause oxidative stress in body tissues. We emphasize that this finding was subsequently confirmed in human tests.^37^ Studies in mice also suggest that combining antioxidant therapy with kinase inhibitors could be used to reduce adverse side effects.^37^ Thus, our systems toxicological approach successfully predicted the molecular mechanisms underlying clinically adverse reactions associated with a given drug.

### Summary of research achievements of this project

Scientific achievements of this project have been published in more than 800 papers in 5 years. Fifty-nine research papers of those are achieved by the joint research between researchers in this project with this project as the first opportunity (by June 2015). This obviously indicates that the core team of project managers had exercised great care in the communication and cooperation between researchers from the different axes, and that it has gone well. Without help from the researchers from the different axes, the first-axis researcher had never developed the research platform with current versatility and usability, while the researcher working on cardiology or small molecular dynamics in the second and third-axes had never tested his/her hypothesis about the functional processes emerged from the complex multilevel biological systems without helps in other axes. Models successfully guided experiments and clinical treatment strategy, and predicted a previously unknown mechanism about several issues in cardiology and small molecular dynamics.

## Influences

Although the HD Physiology Project was completed in 2016, it has already had a tremendous impact. Efforts have already been made to make this platform the global standard. For example, Flint K3, a cloud-based simulation service, was introduced on the NeCTAR research cloud and opened to all Australian researchers, and PhysioDesigner and Flint were introduced on the International Neuroinformatics Coordinating Facility Japan-node Simulation platform operated by Riken NIJC. In Singapore, the Garuda Platform was implemented on the super computer introduced to the A*STAR Computing Resource Center. In addition, there several pharmaceutical companies and cosmetics manufacturers have made inquiries about PhysioDesigner/Flint and the Garuda Platform. The platform’s use and applications are also spreading to the field of education. PhysioDesigner and Flint have been introduced for educational purposes to some national universities in Japan, such as Osaka University and the Kyushu Institute of Technology. That is how use of the platform developed is spreading not only to academia, but also to industry and education.

Moreover, this project has certainly promoted research activities related to systems biology and the physiome in Japan, and has ensured further development in this field. Researchers involved in this project, especially young researchers, have been encouraged and recruited to positions where they can utilize their abilities to the fullest. Several research projects derived from the HD Physiology Project have been conducted, including projects related to translational research that extrapolate the findings of the original investigations and achievements of the HD Physiology Project to the broad fields of laboratory, clinical, and public health research.

## Self-evaluation and outside evaluation of the management of HD Physiology

After the experience in this HD Physiology Project, we affirm that a consortium approach is quite effective in promoting multilevel systems biology and other emerging multidisciplinary research areas. Project leaders need to consider the organic linkage of researchers, and the framework of the organization of the consortium is one of the most important factors. The three-axes framework of HD Physiology Project had worked very well, the researcher in each axis develops his/her research in a synergistic or cooperative manner in the project and more easily in the subdivided axis. Our survey indicates that one subgroup had been collaborating on the subproject with two other subgroups on average (about 80 collaborative subprojects/40 subgroups), and 59 of the research papers have been reported from the collaboration research, as mentioned previously. The size of this project was not too large to manage, but a little smaller size would be better to focus on the most important issues and to clarify the sharing of responsibility and processing execution by persons responsible for each action. The HD Physiology Project made a commitment to promote multilevel integrative systems biology and physiome research, especially in Japan, and had embodied that for 5 years. Achievement of the project was evaluated by an outside reviewing team appointed by MEXT Japan and is appreciated very highly in the aspects of scientific achievements, young researcher development, world-wide leadership, and project management.

## Conclusion

The HD Physiology Project aimed to expand the research field of systems biology to multilevel systems biology in Japan. The software platform that was developed as a result was launched into a public research network, promoting the sustained advancement of the integrative life sciences. The fruits of the HD Physiology Project represents are set to demonstrate the impact and utility of software and tools, and have also addressed many challenges in the fields of cardiology and pharmacokinetics. The tools and protocols that were developed can be applied to other systems and are helping to drive the application of modeling and simulations to medicine. As such, this project has contributed significantly to the development of a new paradigm in biology and medicine. Moreover, qualitative logics and dynamic modeling have the potential to revolutionize medicine by spurring “predictive medicine” based on a vast body of knowledge; in particular, they can be applied to drug discovery and therapy, including personalized medicine, novel diagnostic and surgical procedures, medical device design and use, and education and training. Furthermore, the researchers who participated in this project will lead decision-making regarding a framework to enable interoperability between the different simulation software systems for physiology and clinical purposes. This will be important for future international initiatives for the intellectual processes concerning human life and welfare.
